# 1-[4-(4-Nitro­phen­yl)piperazin-1-yl]-2-(4,5,6,7-tetra­hydro­thieno[3,2-*c*]pyridin-5-yl)ethanone

**DOI:** 10.1107/S1600536810039085

**Published:** 2010-10-09

**Authors:** Shuai Mu, Miao Yang, Deng-Ke Liu, Chang-Xiao Liu

**Affiliations:** a School of Chemical Engineering and Technology, Tianjin University, Tianjin 300072, People’s Republic of China; b Tianjin Medical University, Tianjin 300070, People’s Republic of China; c Tianjin Institute of Pharmaceutical Research, Tianjin 300193, People’s Republic of China

## Abstract

The title compound, C_19_H_22_N_4_O_3_S, comprises a thienopyridine moiety which is characteristic for anti­platelet agents of the clopidogrel class of compounds. In the crystal, inversion dimers are formed through pairs of C—H⋯O inter­actions. The benzene ring plane and the nitro plane are almost coplanar, with a dihedral angle of 0.83 (2)°. The piperazine ring adopts a chair conformation.

## Related literature

For background to the bioactivity and applications of the anti­platelet agent clopidogrel, see, for example: Muller *et al.* (2003 ▶); Savi *et al.* (1994 ▶); Sharis *et al.* (1998 ▶). For the synthesis of other derivatives with thienopyridine, see: Cheng (2009 ▶).
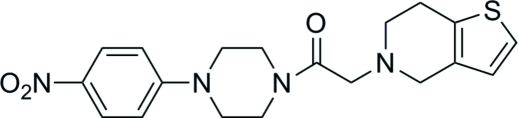


## Experimental

### 

#### Crystal data

C_19_H_22_N_4_O_3_S
*M* *_r_* = 386.47
Triclinic, 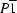

*a* = 6.1315 (7) Å
*b* = 8.8552 (10) Å
*c* = 17.025 (2) Å
α = 84.101 (8)°
β = 83.385 (9)°
γ = 74.635 (6)°
*V* = 882.87 (18) Å^3^
*Z* = 2
Mo *K*α radiation
μ = 0.21 mm^−1^
*T* = 113 K
0.32 × 0.30 × 0.28 mm

#### Data collection

Rigaku Saturn CCD area-detector diffractometer
Absorption correction: multi-scan (*CrystalClear*; Rigaku/MSC, 2005 ▶) *T*_min_ = 0.935, *T*_max_ = 0.943
10552 measured reflections
4169 independent reflections
3402 reflections with *I* > 2σ(*I*)
*R*_int_ = 0.025

#### Refinement

*R*[*F* ^2^ > 2σ(*F* ^2^)] = 0.032
*wR*(*F* ^2^) = 0.088
*S* = 1.08
4169 reflections
245 parameters
H-atom parameters constrained
Δρ_max_ = 0.31 e Å^−3^
Δρ_min_ = −0.26 e Å^−3^

### 

Data collection: *CrystalClear* (Rigaku/MSC, 2005 ▶); cell refinement: *CrystalClear*; data reduction: *CrystalClear*; program(s) used to solve structure: *SHELXS97* (Sheldrick, 2008 ▶); program(s) used to refine structure: *SHELXL97* (Sheldrick, 2008 ▶); molecular graphics: *SHELXL97*; software used to prepare material for publication: *CrystalStructure* (Rigaku/MSC, 2005 ▶).

## Supplementary Material

Crystal structure: contains datablocks global, I. DOI: 10.1107/S1600536810039085/kp2278sup1.cif

Structure factors: contains datablocks I. DOI: 10.1107/S1600536810039085/kp2278Isup2.hkl

Additional supplementary materials:  crystallographic information; 3D view; checkCIF report

## Figures and Tables

**Table 1:** Hydrogen-bond geometry (Å, °)

*D*—H⋯*A*	*D*—H	H⋯*A*	*D*⋯*A*	*D*—H⋯*A*
C1—H1⋯O3^i^	0.95	2.47	3.346 (2)	154
C5—H5*A*⋯O1^ii^	0.99	2.56	3.475 (2)	153
C6—H6*B*⋯O1^iii^	0.99	2.59	3.420 (2)	142

Symmetry codes: (i) 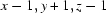; (ii) 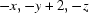; (iii) 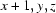.

**Fig. 1.**
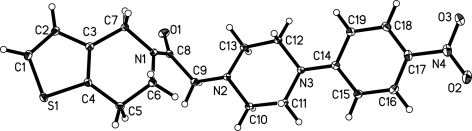
The molecular structure of (I), Displacement ellipsoids are drawn at the 50% probability level.

## supplementary crystallographic information

### Comment

Clopidogrel is an oral, thienopyridine class antiplatelet agent used to inhibit blood clots in coronary artery disease, peripheral vascular disease, and cerebrovascular disease (Muller *et al.*, 2003; Savi *et al.*, 1994; Sharis *et al.*, 1998). The crystal structure of the title compound (I), a derivative with thienopyridine, synthesised through the transformation of clopidogrel, is reported here.

The C14–C19 benzene ring plane and the nitro plane defined by O2/O3/N4 are almost coplanar, with a dihedral angle of 0.83° (Fig. 1). The piperazine ring shows a stable chair conformation. The bond angle in the ring ranges from 107.24–112.67°. The dihedrals formed between C10–C13 plane and C11/C12/N3 plane, C10–C13 plane and C10/C13/N2 plane are 43.32° and 55.40°, respectively. The packing is realised by C—H···O (Table 1) interactions leading to centrosymmetric dimers.

### Experimental

2-Chloro-1-(4-(4-nitrophenyl)piperazin-1-yl)ethanone 4 g (0.014 mol) and anhydrous K_2_CO_3_ 7.7 g (0.056 mol) were dissolved in 40 ml toluene. The mixture was heated to 373 K. Then 2.2 g (0.015 mol) of 4,5,6,7-tetrahydrothieno[3,2-*c*] pyridine was added dropwise into the mixture, and stirred for 16 h under room temperature. K_2_CO_3_ was removed after filtration and the reaction solution was concentrated under reduced pressure to get yellow powder as a crude product. The powder was dissolved in a mixture of petroleum ether (20 ml) and acetone (4 ml) at 277 K, then white crystals were grown slowly.

### Refinement

All the H atoms were located on their parent atoms with C—H = 0.95 Å (aromatic CH) and 0.99 Å (CH2), *U*_iso_ = 1.2*U*_eq_(C).

### Crystal data

**Table:**

C_19_H_22_N_4_O_3_S	*Z* = 2
*M**_r_* = 386.47	*F*(000) = 408
Triclinic, *P*1	*D*_x_ = 1.454 Mg m^−^^3^
*a* = 6.1315 (7) Å	Mo *K*α radiation, λ = 0.71070 Å
*b* = 8.8552 (10) Å	Cell parameters from 2732 reflections
*c* = 17.025 (2) Å	θ = 1.2–27.9°
α = 84.101 (8)°	µ = 0.21 mm^−^^1^
β = 83.385 (9)°	*T* = 113 K
γ = 74.635 (6)°	Block, yellow
*V* = 882.87 (18) Å^3^	0.32 × 0.30 × 0.28 mm

### Data collection

**Table:**

Rigaku Saturn CCD area-detector diffractometer	4169 independent reflections
Radiation source: rotating anode	3402 reflections with *I* > 2σ(*I*)
confocal	*R*_int_ = 0.025
Detector resolution: 7.31 pixels mm^-1^	θ_max_ = 27.9°, θ_min_ = 1.2°
ω and φ scans	*h* = −8→8
Absorption correction: multi-scan (*CrystalClear*; Rigaku/MSC, 2005)	*k* = −11→11
*T*_min_ = 0.935, *T*_max_ = 0.943	*l* = −22→21
10552 measured reflections	

### Refinement

**Table:**

Refinement on *F*^2^	Secondary atom site location: difference Fourier map
Least-squares matrix: full	Hydrogen site location: inferred from neighbouring sites
*R*[*F*^2^ > 2σ(*F*^2^)] = 0.032	H-atom parameters constrained
*wR*(*F*^2^) = 0.088	*w* = 1/[σ^2^(*F*_o_^2^) + (0.0455*P*)^2^ + 0.1626*P*] where *P* = (*F*_o_^2^ + 2*F*_c_^2^)/3
*S* = 1.08	(Δ/σ)_max_ = 0.001
4169 reflections	Δρ_max_ = 0.31 e Å^−^^3^
245 parameters	Δρ_min_ = −0.26 e Å^−^^3^
0 restraints	Extinction correction: *SHELXL97* (Sheldrick, 2008), Fc^*^=kFc[1+0.001xFc^2^λ^3^/sin(2θ)]^-1/4^
Primary atom site location: structure-invariant direct methods	Extinction coefficient: 0.019 (7)

### Special details

**Table:**

Geometry. All e.s.d.'s (except the e.s.d. in the dihedral angle between two l.s. planes) are estimated using the full covariance matrix. The cell e.s.d.'s are taken into account individually in the estimation of e.s.d.'s in distances, angles and torsion angles; correlations between e.s.d.'s in cell parameters are only used when they are defined by crystal symmetry. An approximate (isotropic) treatment of cell e.s.d.'s is used for estimating e.s.d.'s involving l.s. planes.
Refinement. Refinement of *F*^2^ against ALL reflections. The weighted *R*-factor *wR* and goodness of fit *S* are based on *F*^2^, conventional *R*-factors *R* are based on *F*, with *F* set to zero for negative *F*^2^. The threshold expression of *F*^2^ > σ(*F*^2^) is used only for calculating *R*-factors(gt) *etc*. and is not relevant to the choice of reflections for refinement. *R*-factors based on *F*^2^ are statistically about twice as large as those based on *F*, and *R*- factors based on ALL data will be even larger.

### Fractional atomic coordinates and isotropic or equivalent isotropic displacement parameters (Å^2^)

**Table:**

	*x*	*y*	*z*	*U*_iso_*/*U*_eq_	
S1	0.30943 (5)	0.92591 (3)	−0.249539 (17)	0.01923 (11)	
O1	−0.12392 (14)	0.78988 (11)	0.05647 (6)	0.0249 (2)	
O2	1.01128 (18)	0.15750 (13)	0.58507 (6)	0.0376 (3)	
O3	0.7589 (2)	0.02733 (13)	0.58411 (7)	0.0451 (3)	
N1	0.22099 (16)	0.69145 (11)	−0.00851 (6)	0.0145 (2)	
N2	0.32167 (16)	0.71481 (11)	0.16167 (6)	0.0148 (2)	
N3	0.44060 (16)	0.56269 (11)	0.31518 (6)	0.0156 (2)	
N4	0.8425 (2)	0.13723 (14)	0.55919 (7)	0.0275 (3)	
C1	0.0996 (2)	0.85073 (14)	−0.27522 (7)	0.0208 (3)	
H1	0.0370	0.8743	−0.3249	0.025*	
C2	0.0340 (2)	0.75339 (14)	−0.21584 (7)	0.0183 (2)	
H2	−0.0804	0.7006	−0.2193	0.022*	
C3	0.15476 (19)	0.73858 (13)	−0.14760 (7)	0.0147 (2)	
C4	0.31018 (19)	0.82610 (13)	−0.15692 (7)	0.0147 (2)	
C5	0.45773 (19)	0.83687 (14)	−0.09431 (7)	0.0162 (2)	
H5A	0.3991	0.9385	−0.0700	0.019*	
H5B	0.6148	0.8302	−0.1181	0.019*	
C6	0.45572 (19)	0.70088 (13)	−0.03118 (7)	0.0156 (2)	
H6A	0.5467	0.6011	−0.0523	0.019*	
H6B	0.5245	0.7176	0.0160	0.019*	
C7	0.1199 (2)	0.64098 (14)	−0.07174 (7)	0.0163 (2)	
H7A	−0.0446	0.6535	−0.0570	0.020*	
H7B	0.1916	0.5287	−0.0792	0.020*	
C8	0.08392 (19)	0.77300 (13)	0.04926 (7)	0.0161 (2)	
C9	0.1956 (2)	0.83986 (14)	0.10806 (7)	0.0171 (2)	
H9A	0.3008	0.8981	0.0788	0.021*	
H9B	0.0775	0.9147	0.1396	0.021*	
C10	0.4469 (2)	0.78077 (14)	0.21157 (7)	0.0173 (2)	
H10A	0.3387	0.8607	0.2433	0.021*	
H10B	0.5515	0.8332	0.1775	0.021*	
C11	0.5819 (2)	0.65394 (14)	0.26673 (7)	0.0181 (2)	
H11A	0.7032	0.5820	0.2349	0.022*	
H11B	0.6556	0.7033	0.3022	0.022*	
C12	0.2908 (2)	0.51145 (14)	0.26835 (7)	0.0172 (2)	
H12A	0.1800	0.4685	0.3047	0.021*	
H12B	0.3824	0.4263	0.2358	0.021*	
C13	0.1640 (2)	0.64430 (14)	0.21457 (7)	0.0177 (2)	
H13A	0.0697	0.6039	0.1826	0.021*	
H13B	0.0615	0.7254	0.2471	0.021*	
C14	0.5398 (2)	0.45722 (13)	0.37518 (7)	0.0157 (2)	
C15	0.7310 (2)	0.47506 (14)	0.40870 (7)	0.0194 (3)	
H15	0.7935	0.5608	0.3898	0.023*	
C16	0.8282 (2)	0.37062 (15)	0.46814 (7)	0.0212 (3)	
H16	0.9579	0.3837	0.4895	0.025*	
C17	0.7371 (2)	0.24656 (14)	0.49676 (7)	0.0207 (3)	
C18	0.5485 (2)	0.22540 (14)	0.46630 (7)	0.0214 (3)	
H18	0.4861	0.1404	0.4867	0.026*	
C19	0.4521 (2)	0.32864 (14)	0.40612 (7)	0.0194 (3)	
H19	0.3237	0.3131	0.3849	0.023*	

### Atomic displacement parameters (Å^2^)

**Table:**

	*U*^11^	*U*^22^	*U*^33^	*U*^12^	*U*^13^	*U*^23^
S1	0.02648 (18)	0.01618 (16)	0.01532 (16)	−0.00697 (12)	−0.00135 (12)	0.00086 (11)
O1	0.0145 (4)	0.0317 (5)	0.0297 (5)	−0.0068 (4)	0.0002 (4)	−0.0080 (4)
O2	0.0329 (6)	0.0445 (6)	0.0350 (6)	−0.0089 (5)	−0.0162 (5)	0.0104 (5)
O3	0.0627 (8)	0.0347 (6)	0.0444 (7)	−0.0234 (6)	−0.0272 (6)	0.0207 (5)
N1	0.0127 (5)	0.0165 (5)	0.0148 (5)	−0.0046 (4)	−0.0031 (4)	0.0004 (4)
N2	0.0149 (5)	0.0158 (5)	0.0154 (5)	−0.0068 (4)	−0.0027 (4)	0.0006 (4)
N3	0.0160 (5)	0.0161 (5)	0.0164 (5)	−0.0074 (4)	−0.0027 (4)	0.0005 (4)
N4	0.0324 (6)	0.0252 (6)	0.0230 (6)	−0.0035 (5)	−0.0069 (5)	0.0023 (5)
C1	0.0269 (6)	0.0191 (6)	0.0169 (6)	−0.0045 (5)	−0.0062 (5)	−0.0025 (5)
C2	0.0210 (6)	0.0172 (5)	0.0179 (6)	−0.0049 (5)	−0.0042 (5)	−0.0045 (5)
C3	0.0148 (5)	0.0117 (5)	0.0166 (6)	−0.0014 (4)	−0.0016 (4)	−0.0022 (4)
C4	0.0162 (5)	0.0121 (5)	0.0148 (5)	−0.0021 (4)	−0.0010 (4)	−0.0009 (4)
C5	0.0141 (5)	0.0165 (5)	0.0187 (6)	−0.0057 (4)	−0.0020 (5)	0.0008 (4)
C6	0.0116 (5)	0.0170 (5)	0.0178 (6)	−0.0029 (4)	−0.0033 (4)	0.0013 (4)
C7	0.0182 (6)	0.0163 (5)	0.0163 (6)	−0.0071 (4)	−0.0048 (5)	0.0006 (4)
C8	0.0167 (6)	0.0136 (5)	0.0178 (6)	−0.0040 (4)	−0.0037 (5)	0.0020 (4)
C9	0.0185 (6)	0.0149 (5)	0.0184 (6)	−0.0048 (4)	−0.0021 (5)	−0.0011 (4)
C10	0.0191 (6)	0.0174 (5)	0.0183 (6)	−0.0092 (5)	−0.0036 (5)	−0.0006 (5)
C11	0.0168 (6)	0.0203 (6)	0.0199 (6)	−0.0101 (5)	−0.0026 (5)	0.0008 (5)
C12	0.0171 (6)	0.0182 (6)	0.0191 (6)	−0.0096 (5)	−0.0036 (5)	0.0010 (5)
C13	0.0144 (5)	0.0198 (6)	0.0202 (6)	−0.0075 (4)	−0.0016 (5)	0.0004 (5)
C14	0.0166 (5)	0.0151 (5)	0.0148 (5)	−0.0033 (4)	0.0017 (4)	−0.0037 (4)
C15	0.0205 (6)	0.0202 (6)	0.0191 (6)	−0.0076 (5)	−0.0021 (5)	−0.0024 (5)
C16	0.0208 (6)	0.0236 (6)	0.0203 (6)	−0.0060 (5)	−0.0042 (5)	−0.0039 (5)
C17	0.0245 (6)	0.0190 (6)	0.0162 (6)	−0.0012 (5)	−0.0023 (5)	−0.0010 (5)
C18	0.0264 (7)	0.0175 (6)	0.0204 (6)	−0.0069 (5)	−0.0003 (5)	−0.0003 (5)
C19	0.0204 (6)	0.0190 (6)	0.0199 (6)	−0.0070 (5)	−0.0025 (5)	−0.0015 (5)

### Geometric parameters (Å, °)

**Table:**

S1—C1	1.7127 (13)	C6—H6B	0.9900
S1—C4	1.7265 (12)	C7—H7A	0.9900
O1—C8	1.2362 (14)	C7—H7B	0.9900
O2—N4	1.2317 (16)	C8—C9	1.5219 (16)
O3—N4	1.2327 (16)	C9—H9A	0.9900
N1—C8	1.3524 (16)	C9—H9B	0.9900
N1—C7	1.4638 (14)	C10—C11	1.5160 (16)
N1—C6	1.4685 (14)	C10—H10A	0.9900
N2—C13	1.4628 (14)	C10—H10B	0.9900
N2—C10	1.4637 (14)	C11—H11A	0.9900
N2—C9	1.4690 (15)	C11—H11B	0.9900
N3—C14	1.3900 (15)	C12—C13	1.5115 (16)
N3—C12	1.4662 (14)	C12—H12A	0.9900
N3—C11	1.4674 (14)	C12—H12B	0.9900
N4—C17	1.4491 (16)	C13—H13A	0.9900
C1—C2	1.3558 (18)	C13—H13B	0.9900
C1—H1	0.9500	C14—C15	1.4125 (17)
C2—C3	1.4251 (16)	C14—C19	1.4140 (16)
C2—H2	0.9500	C15—C16	1.3746 (17)
C3—C4	1.3658 (16)	C15—H15	0.9500
C3—C7	1.5051 (16)	C16—C17	1.3818 (18)
C4—C5	1.5008 (16)	C16—H16	0.9500
C5—C6	1.5311 (16)	C17—C18	1.3833 (18)
C5—H5A	0.9900	C18—C19	1.3768 (17)
C5—H5B	0.9900	C18—H18	0.9500
C6—H6A	0.9900	C19—H19	0.9500
			
C1—S1—C4	92.11 (6)	N2—C9—H9A	109.4
C8—N1—C7	119.36 (10)	C8—C9—H9A	109.4
C8—N1—C6	123.35 (10)	N2—C9—H9B	109.4
C7—N1—C6	113.03 (9)	C8—C9—H9B	109.4
C13—N2—C10	107.24 (9)	H9A—C9—H9B	108.0
C13—N2—C9	110.12 (9)	N2—C10—C11	111.11 (9)
C10—N2—C9	109.88 (9)	N2—C10—H10A	109.4
C14—N3—C12	117.17 (9)	C11—C10—H10A	109.4
C14—N3—C11	117.63 (9)	N2—C10—H10B	109.4
C12—N3—C11	112.57 (9)	C11—C10—H10B	109.4
O2—N4—O3	122.75 (11)	H10A—C10—H10B	108.0
O2—N4—C17	118.95 (11)	N3—C11—C10	112.67 (10)
O3—N4—C17	118.30 (11)	N3—C11—H11A	109.1
C2—C1—S1	111.59 (9)	C10—C11—H11A	109.1
C2—C1—H1	124.2	N3—C11—H11B	109.1
S1—C1—H1	124.2	C10—C11—H11B	109.1
C1—C2—C3	112.85 (11)	H11A—C11—H11B	107.8
C1—C2—H2	123.6	N3—C12—C13	112.06 (9)
C3—C2—H2	123.6	N3—C12—H12A	109.2
C4—C3—C2	112.58 (11)	C13—C12—H12A	109.2
C4—C3—C7	121.64 (10)	N3—C12—H12B	109.2
C2—C3—C7	125.77 (10)	C13—C12—H12B	109.2
C3—C4—C5	124.49 (11)	H12A—C12—H12B	107.9
C3—C4—S1	110.87 (9)	N2—C13—C12	110.96 (9)
C5—C4—S1	124.63 (9)	N2—C13—H13A	109.4
C4—C5—C6	108.25 (9)	C12—C13—H13A	109.4
C4—C5—H5A	110.0	N2—C13—H13B	109.4
C6—C5—H5A	110.0	C12—C13—H13B	109.4
C4—C5—H5B	110.0	H13A—C13—H13B	108.0
C6—C5—H5B	110.0	N3—C14—C15	121.43 (11)
H5A—C5—H5B	108.4	N3—C14—C19	121.51 (11)
N1—C6—C5	109.67 (9)	C15—C14—C19	117.06 (11)
N1—C6—H6A	109.7	C16—C15—C14	121.21 (11)
C5—C6—H6A	109.7	C16—C15—H15	119.4
N1—C6—H6B	109.7	C14—C15—H15	119.4
C5—C6—H6B	109.7	C15—C16—C17	119.90 (12)
H6A—C6—H6B	108.2	C15—C16—H16	120.0
N1—C7—C3	109.44 (9)	C17—C16—H16	120.0
N1—C7—H7A	109.8	C16—C17—C18	120.89 (12)
C3—C7—H7A	109.8	C16—C17—N4	119.13 (12)
N1—C7—H7B	109.8	C18—C17—N4	119.98 (11)
C3—C7—H7B	109.8	C19—C18—C17	119.43 (11)
H7A—C7—H7B	108.2	C19—C18—H18	120.3
O1—C8—N1	122.19 (11)	C17—C18—H18	120.3
O1—C8—C9	120.27 (11)	C18—C19—C14	121.51 (11)
N1—C8—C9	117.51 (10)	C18—C19—H19	119.2
N2—C9—C8	111.26 (9)	C14—C19—H19	119.2
			
C4—S1—C1—C2	−0.24 (10)	C9—N2—C10—C11	−179.10 (9)
S1—C1—C2—C3	0.11 (14)	C14—N3—C11—C10	−171.71 (10)
C1—C2—C3—C4	0.12 (15)	C12—N3—C11—C10	47.41 (13)
C1—C2—C3—C7	179.31 (11)	N2—C10—C11—N3	−54.79 (13)
C2—C3—C4—C5	178.37 (10)	C14—N3—C12—C13	170.61 (10)
C7—C3—C4—C5	−0.85 (17)	C11—N3—C12—C13	−48.32 (13)
C2—C3—C4—S1	−0.30 (13)	C10—N2—C13—C12	−62.45 (12)
C7—C3—C4—S1	−179.52 (9)	C9—N2—C13—C12	178.02 (9)
C1—S1—C4—C3	0.31 (9)	N3—C12—C13—N2	56.93 (13)
C1—S1—C4—C5	−178.36 (10)	C12—N3—C14—C15	162.26 (10)
C3—C4—C5—C6	16.48 (15)	C11—N3—C14—C15	23.17 (16)
S1—C4—C5—C6	−165.03 (8)	C12—N3—C14—C19	−18.64 (16)
C8—N1—C6—C5	−87.64 (13)	C11—N3—C14—C19	−157.73 (11)
C7—N1—C6—C5	68.93 (12)	N3—C14—C15—C16	179.86 (11)
C4—C5—C6—N1	−47.59 (12)	C19—C14—C15—C16	0.72 (18)
C8—N1—C7—C3	107.55 (12)	C14—C15—C16—C17	−0.80 (19)
C6—N1—C7—C3	−50.05 (12)	C15—C16—C17—C18	0.11 (19)
C4—C3—C7—N1	16.24 (15)	C15—C16—C17—N4	179.73 (11)
C2—C3—C7—N1	−162.88 (11)	O2—N4—C17—C16	−0.17 (18)
C7—N1—C8—O1	9.29 (17)	O3—N4—C17—C16	179.92 (13)
C6—N1—C8—O1	164.47 (11)	O2—N4—C17—C18	179.45 (12)
C7—N1—C8—C9	−172.84 (9)	O3—N4—C17—C18	−0.46 (19)
C6—N1—C8—C9	−17.67 (16)	C16—C17—C18—C19	0.64 (19)
C13—N2—C9—C8	−68.22 (12)	N4—C17—C18—C19	−178.97 (11)
C10—N2—C9—C8	173.88 (9)	C17—C18—C19—C14	−0.72 (19)
O1—C8—C9—N2	106.91 (12)	N3—C14—C19—C18	−179.10 (11)
N1—C8—C9—N2	−71.00 (13)	C15—C14—C19—C18	0.05 (18)
C13—N2—C10—C11	61.22 (12)		

### Hydrogen-bond geometry (Å, °)

**Table:**

*D*—H···*A*	*D*—H	H···*A*	*D*···*A*	*D*—H···*A*
C1—H1···O3^i^	0.95	2.47	3.346 (2)	154
C5—H5A···O1^ii^	0.99	2.56	3.475 (2)	153
C6—H6B···O1^iii^	0.99	2.59	3.420 (2)	142

Symmetry codes: (i) *x*−1, *y*+1, *z*−1; (ii) −*x*, −*y*+2, −*z*; (iii) *x*+1, *y*, *z*.
